# Neighborhood social cohesion and serious psychological distress among Asian, Black, Hispanic/Latinx, and White adults in the United States: a cross-sectional study

**DOI:** 10.1186/s12889-022-13572-4

**Published:** 2022-06-15

**Authors:** Lauren R. Gullett, Dana M. Alhasan, Symielle A. Gaston, W. Braxton Jackson, Ichiro Kawachi, Chandra L. Jackson

**Affiliations:** 1 Epidemiology Branch, National Institute of Environmental Health Sciences, National Institutes of Health, Department of Health and Human Services, 111 TW Alexander Drive, MD A3-05, Research Triangle Park, NC 27709 USA; 2 Social and Scientific Systems, Inc., a DLH Holdings Company, NC Durham, USA; 3 Department of Social and Behavioral Sciences, Harvard T.H. Chan School of Public Health, Boston, MA USA; 4 Intramural Program, National Institute on Minority Health and Health Disparities, National Institutes of Health, Department of Health and Human Services, Bethesda, MD USA

**Keywords:** Residence characteristics, Community support, Psychological distress, Mental health, Race factors, Economic status

## Abstract

**Background:**

Serious psychological distress (SPD) is common and more prevalent in women, older adults, and individuals with a low-income. Prior studies have highlighted the role of low neighborhood social cohesion (nSC) in potentially contributing to SPD; however, few have investigated this association in a large, nationally representative sample of the United States. Therefore, our objective was to investigate the overall and racial/ethnic-, sex/gender-, self-rated health status-, age-, and household income-specific relationships between nSC and SPD.

**Methods:**

We used data from survey years 2013 to 2018 of the National Health Interview Survey to investigate nSC and SPD among Asian, Non-Hispanic (NH)-Black, Hispanic/Latinx, and NH-White men as well as women in the United States (*N* = 168,573) and to determine modification by race/ethnicity, sex/gender, self-rated health status, age, and annual household income. nSC was measured by asking participants four questions related to the trustworthiness and dependability of their neighbors. nSC scores were trichotomized into low (< 12), medium (12–14), and high (15–16). SPD was measured using the Kessler 6 psychological distress scale with scores ≥ 13 indicating SPD. After adjusting for sociodemographic, health behavior, and clinical confounders, we used Poisson regression with robust variance to estimate prevalence ratios (PRs) and 95% confidence intervals (CIs).

**Results:**

Among 168,573 participants, most were Non-Hispanic (NH)-White (69%), and mean age was 47 ± 0.01 years. After adjustment, low vs. high nSC was associated with a 75% higher prevalence of SPD overall (PR = 1.75 [1.59–1.92]), 4 times the prevalence of SPD among Asian men (PR = 4.06 [1.57–10.50]), 2 times the prevalence of SPD among participants in at least good health (PR = 2.02 [95% CI: 1.74–2.35]), 92% higher prevalence of SPD among participants ≥ 50 years old (PR = 1.92 [1.70–2.18]), and approximately 3 times the prevalence of SPD among Hispanic/Latinx participants with household incomes ≥ $75,000 (PR = 2.97 [1.45–6.08]).

**Conclusions:**

Low nSC was associated with higher SPD in the overall population and the magnitude of the association was higher in Asian men, participants who reported good health, older participants, and Hispanic/Latinx adults with higher household incomes. Future research should continue to examine how neighborhood contexts can affect health across various sociodemographic groups, especially among groups with multiple marginalized social identities.

**Supplementary Information:**

The online version contains supplementary material available at 10.1186/s12889-022-13572-4.

## Background

Serious psychological distress (SPD), which impacts approximately 10 million adults in the United States (US), comprises a range of emotions and mood disorders (e.g., depression, anxiety, nervousness, sadness, irritability) that can impede one’s ability to effectively respond to everyday demands of life [1–4]. The prevalence of SPD is higher among Hispanic/Latinx and Non-Hispanic (NH)-Black individuals, women, individuals with physical health conditions (e.g., heart disease), older adults, and adults with lower income compared to NH-White individuals, men, individuals without physical health conditions, younger adults, and adults with higher income, respectively [4, 5]. Moreover, SPD has been associated with and may contribute to adverse health behaviors and outcomes, such as arthritis, type 2 diabetes, and cardiovascular disease [4]. Consequently, it is important to identify potential modifiable determinants of SPD to inform intervention strategies.

Prior studies highlighted adverse socio-environmental exposures, including lack of neighborhood social cohesion (nSC), as potential contributors to SPD [6–8]. nSC is considered the level of interconnectedness, solidarity, mutual trust, and shared values among neighbors [9] and may influence SPD through sociological, environmental, behavioral, and biological pathways. Low nSC may involve decreased feelings of safety, trust, and social support; increased demoralization; and may reinforce unhealthy social norms, such as alcohol consumption [10–12]. This, in turn, is hypothesized to activate the hypothalamic–pituitary–adrenal (HPA) axis, the body’s central stress response system [11, 13–16], where stress can lead to SPD [11, 13–17]. Conversely, living in a neighborhood with high levels of social cohesion may increase feelings of safety, trust, and social support as well as reinforce healthy behaviors, including sufficient sleep [18, 19]. These positive experiences and healthy behaviors can help alleviate stress and, thus, contribute to better mental health outcomes [20].

The relationship between nSC and SPD may be modified by influential characteristics including race/ethnicity, sex/gender, self-rated health status, age, and household income. For instance, the neighborhoods of minoritized racial/ethnic groups are often characterized by poorly built infrastructure, neglectful environmental standards, and fewer resources due to historical and contemporary forms of racial residential segregation resulting from structural racism [21–23]. These differential exposures across race/ethnicity can lead to differences in perceived nSC by race/ethnicity. Furthermore, women may perceive aspects of nSC differently than men, as women may be more influenced by socioenvironmental factors such as safety, social support, and sociocultural norms [24–26]. Individuals with poor self-rated health generally experience barriers related to interacting with members of their community, which may influence their perception of nSC compared to individuals with good self-rated health (e.g., individuals with arthritis are less likely to use their neighborhood as a means to meet physical activity guidelines, a common way to interact with community members) [27–29]. However, a prior study on sleep health and SPD showed that the association between poor sleep and SPD was stronger among adults with good versus poor self-rated health [30]. The authors suggested that adults with good versus poor health were less likely to have exposures that obscure the sleep-SPD relationship. This phenomenon may also exist in the nSC-SPD relationship, which warrants investigation [30]. Since older adults – who are typically less mobile – tend to be more dependent on the material and social resources (e.g., easily accessible community centers [31]) in their immediate surroundings, this age group may be more strongly influenced by nSC than younger adults [32]. This was previously demonstrated by a US study that found adults > 50 years old who perceived high neighborhood social cohesion had fewer outcomes related to psychological distress and better wellbeing outcomes [33]. Lastly, due to persistent socioeconomic inequity [34], people with lower household incomes are more likely to live in poorer neighborhoods with fewer resources, lower quality housing, and more environmental hazards [26, 35], which likely impacts levels of perceived social ties and support, and could subsequently influence perceived nSC [36]. These potential sociodemographic differences in the pathways from nSC to SPD are grounded in the socioecological framework, which asserts that nSC is influenced by upstream, societal drivers like structural racism [37].

It is important to examine the nSC-SPD relationship on a national scale and among a large racially/ethnically diverse sample of the US population since previous studies examining the nSC-SPD relationship have mostly been conducted outside of the US (e.g., Canada, United Kingdom), have rarely considered diverse racial/ethnic groups beyond White populations, and had small sample sizes [6, 18–20, 38–40]. Therefore, the objective of this study was to investigate the relationship between nSC and SPD overall and – given the potential to modify the association – by race/ethnicity, sex/gender, self-rated health status, age, and household income using nationally representative data from the National Health Interview Survey (NHIS). We hypothesized that low and medium compared to high nSC would be associated with a higher prevalence of SPD. We also hypothesized that the relationship between nSC and SPD would differ by race/ethnicity, sex/gender, self-rated health status, age, and household income in that – at the same level of nSC – a higher prevalence of SPD will be observed among minoritized racial/ethnic groups compared to Whites, women compared to men, participants in good compared to poor health, older compared to younger adults, and participants with lower compared to higher household incomes. Additionally, we hypothesized that groups with more than one marginalized social identity (e.g., Black and women) would have a higher prevalence of SPD than groups with one or no marginalized social identity.

## Methods

### Study design

This cross-sectional study examined the relationship between low and medium vs. high nSC and SPD in a large sample of US adults, overall, as well as in groups stratified by race/ethnicity, sex/gender, self-rated health status, age, and annual household income. Data used in this study were from years 2013 to 2018 of the National Health Interview Survey, which were pooled by the Integrated Health Interview Series [41].

### Data source

The NHIS is a cross-sectional, nationwide survey that has collected information about the health of the US civilian non-institutionalized population since 1957 [42]. The NHIS, conducted by the National Center for Health Statistics and the Centers for Disease Control and Prevention, employs a multistage stratified sampling technique to select a representative sample of the US population annually. Detailed study protocol is described elsewhere [42]. Briefly, personnel from the US Census Bureau conduct voluntary, face-to-face computer-assisted household interviews about the health of the participants. The overall sample adult response rate was 56.1% (range: 61.2% (2013)—53.1% (2018)). We used sampling weights to account for the survey’s complex sampling design, non-response, and oversampling of certain groups (e.g., minoritized racial/ethnic groups; elderly). Participants provided informed consent, and the National Institute of Environmental Health Sciences Institutional Review Board waived approval for publicly available, secondary data with no identifiable information.

### Study population

The analysis included participants ≥ 18 years of age. Of the 190,113 who were interviewed, we excluded participants with missing information on nSC (*n* = 14,327), SPD (*n* = 2,120), and race/ethnicity (*n* = 361) (Supplemental Fig. 1). Native Americans (*n* = 1,481) and multiple additional racial/ethnic groups (*n *= 3,251) were excluded due to a small sample size. Therefore, the final analytic sample comprised 168,573 participants.

### Exposure assessment: neighborhood social cohesion

nSC was defined using questions adapted from the Project on Human Development in Chicago Neighborhoods [43, 44]. Participants were asked to respond to the following four statements about how they perceive their neighborhood: (1) this is a close-knit neighborhood, (2) there are people I can count on in this neighborhood, (3) people in this neighborhood can be trusted, and (4) people in this neighborhood help each other out. Responses were measured on a four-point Likert scale: (1) strongly disagree, (2) somewhat disagree, (3) somewhat agree, and (4) strongly agree. Scores were summed and ranged from 4 to 16. Consistent with prior literature [44, 45], nSC was trichotomized into the following categories: low (< 12), medium (12–14), and high (15–16).

### Outcome assessment: serious psychological distress

SPD was measured using the Kessler Psychological Distress Scale (K6) [46], which is a validated and frequently used screening tool for serious mental illness that has high specificity across racial/ethnic groups [46]. Participants were asked how often they felt the following during the past 30 days: (1) nervous, (2) restless/fidgety, (3) hopeless, (4) so sad that nothing could cheer you up, (5) worthless, and (6) everything was an effort. Responses were measured on a five-point Likert scale: (0) none of the time, (1) a little of the time, (2) some of the time, (3) most of the time, and (4) all of the time. Scores were summed and ranged from 0 to 24. Higher scores represented higher levels of SPD, which was dichotomized as no SPD (< 13) and SPD (≥ 13) to be consistent with the evidence-based cut-points determined by prior literature [46].

### Potential confounders

All potential confounders were determined a priori and were self-reported. The following sociodemographic characteristics were considered confounders: age (18–30, 31–49, or ≥ 50 years), sex/gender (women or men), race/ethnicity (Asian, NH-Black, Hispanic/Latinx, and NH-White), marital status (married/living with partner/cohabitating, divorced/widowed/separated, or single/no live-in partner), educational attainment (< high school, high school graduate, some college, or ≥ college), annual household income (< $35,000, $35,000-$74,999, or ≥ $75,000), occupational class (professional/management, support services, or laborers), region of residence (Northeast, Midwest, South, or West), and employment status (unemployed/not in the labor force or employed). We considered the following health behaviors confounders: smoking status (never smoking/quit > 12 months prior, quit ≤ 12 months ago, or current), leisure-time physical activity (PA) based on recommended guidelines of ≥ 150 min/week of moderate intensity or ≥ 75 min/week of vigorous intensity [47] (never/unable, does not meet PA guidelines, or meets PA guidelines), and alcohol consumption status (never, former, or current). We considered the following clinical characteristics confounders: body mass index (BMI) (< 18.5 kg/m^2^ (underweight), 18.5- < 25 kg/m^2^ (recommended), 25–29.9 kg/m^2^ (overweight), or ≥ 30 kg/m^2^ (obese)), self-rated health status (excellent/very good/good or fair/poor) as well as a prior diagnosis (yes or no) of the following: dyslipidemia, hypertension, and prediabetes/diabetes. Rather than considering each health behavior and clinical characteristic separately, “ideal” cardiovascular health (yes or no) was determined based on participants meeting all of the following criteria: never smoking/quit > 12 months prior to interview, meeting leisure-time PA guidelines, BMI 18.5- < 25 kg/m^2^, and no prior diagnosis of dyslipidemia, hypertension, or diabetes/prediabetes [48].

### Potential modifiers: race/ethnicity, sex/gender, self-rated health status, age, and annual household income

We investigated the following characteristics as potential modifiers of the nSC-SPD relationship: race/ethnicity, sex/gender, health status, age, and household income [22, 25, 33, 34, 49].

### Statistical analyses

We calculated descriptive statistics and presented continuous variables as means ± standard errors (S.E.) and categorical variables as percentages that were age-standardized to the 2010 US Census population. Furthermore, we used Poisson regression with robust variance models to directly estimate prevalence ratios (PRs) and 95% confidence intervals (CIs) [50]. We adjusted for the following confounders in the model for the overall study population: age, sex/gender, race/ethnicity, marital status, educational attainment, annual household income, occupational class, region of residence, employment status, alcohol consumption status, self-rated health status, and “ideal” cardiovascular health.

We investigated potential differences in the association between nSC and SPD by race/ethnicity, sex/gender, self-rated health status, age, and annual household income through stratification and formal testing of statistical interaction. We also compared low, medium, and high nSC among minoritized racial/ethnic groups to NH-White participants living in high nSC. We used a two-sided alpha level of 0.05 to determine statistical significance and conducted analyses using Stata version 15 (StataCorp LLC, College Station, TX).

### Sensitivity analyses

Because length of residence within a neighborhood may affect one’s perception of nSC [51], we conducted a sensitivity analysis to estimate associations between nSC and SPD while accounting for length of residence (< 1, 1–10, or ≥ 10 years). We also assessed the robustness of the outcome by comparing the original outcome of SPD from the main analyses (K6 score of ≥ 13) to SPD along with less serious but clinically relevant psychological distress (K6 score of 5–12) [46].

## Results

### Study population characteristics

The mean age of the 168,573 eligible participants was 47 ± 0.1 years and 60.3% of participants were ≥ 50 years old (Table 1). Women comprised 51.9% of the sample and the racial/ethnic composition was 5.4% Asian, 11.2% NH-Black, 14.6% Hispanic/Latinx, and 68.9% NH-White. Most participants had annual household incomes < $75,000 (58.9%) and reported being in excellent/very good/good health (referred to as at least good health) (85.8%). Moreover, 32.0% of participants reported living in a neighborhood with low social cohesion, 33.0% reported medium, and 35.0% high. Hispanic/Latinx and NH-Black participants were overrepresented among those who perceived low nSC and NH-White participants were overrepresented among those in the high nSC category (Table 1).

**Table 1:** Sociodemographic, Health Behavior, and Clinical Characteristics by nSC Level, and Stratified by SPD Status (*N* = 168,573)

	**Low Cohesion** ***n* = 53,964 (32.0%)**			**Medium Cohesion** ***n* = 55,611 (33.0%)**			**High Cohesion** ***n* = 58,998 (35.0%)**			**Total** ***N* = 168,573**		
	**All** ***N* = ** **53,964**	**No SPD** ***N* = 50,717**	**With SPD** ***N* = 3247**	**All** ***N* = ** **55,611**	**No SPD** ***N* = ** **54,016**	**With SPD** ***N* = 1595**	**All** ***N* = ** **58,998**	**No SPD** ***N* = ** **57,541**	**With SPD** ***N* = 1457**	**All** ***N* = ** **168,573**	**No SPD** ***N* = ** **162,274**	**With SPD** ***N* = 6299**
**Serious Psychological Distress**^a^ (yes)	6.0%	–	–	2.9%	–	–	2.5%	–	–	3.7%	–	–
**Age, mean ± SE (years)**	44 ± 0.1	44 ± 0.1	46 ± 0.4	47 ± 0.1	47 ± 0.1	48 ± 0.6	51 ± 0.1	51 ± 0.1	50 ± 0.6	47 ± 0.1	47 ± 0.1	47 ± 0.3
18–30 (%)	18.7	18.8	16.0	16.2	16.2	15.4	13.7	13.7	12.8	16.4	16.4	15.2
31–49 (%)	21.0	20.8	23.7	23.5	23.4	24.3	26.0	25.9	26.8	23.3	23.3	24.5
≥ 50 (%)	60.3	60.3	60.3	60.3	60.3	60.3	60.3	60.3	60.3	60.3	60.3	60.3
**Women (%)**	53.2	52.7	62.5	49.5	49.3	59.1	53.0	52.8	62.7	51.9	51.6	61.7
**Race/ethnicity**												
Asian (%)	5.1	5.3	2.2	6.2	6.3	4.4	4.9	5.0	2.7	5.4	5.5	2.9
Black (%)	14.2	14.3	11.6	11.7	11.7	11.8	7.9	7.8	10.6	11.2	11.1	11.5
Hispanic/Latinx (%)	19.4	19.3	20.6	14.2	14.2	14.6	10.4	10.3	13.9	14.6	14.5	17.5
White (%)	61.3	61.0	65.7	67.9	67.8	69.1	76.8	76.9	72.8	68.9	68.9	68.1
**Marital/Co-habiting status**												
Married/living with partner or cohabitating (%)	55.2	56.0	42.1	62.3	62.7	45.6	66.6	67.1	46.1	61.6	62.2	44.1
Divorced/widowed/no live-In partner (%)	24.0	23.4	34.7	19.8	19.4	33.0	18.5	18.1	33.0	20.5	20.0	33.8
Single/no live-in partner (%)	20.8	20.6	23.2	18.0	17.9	21.4	14.9	14.8	20.9	17.9	17.8	22.1
**Educational attainment**												
< High school (%)	13.7	13.1	23.8	9.5	9.2	22.5	8.3	8.0	21.9	10.4	9.9	23.0
High school graduate (%)	29.9	29.6	34.7	26.5	26.3	32.4	26.1	25.8	35.2	27.5	27.2	34.1
Some college (%)	31.1	31.1	30.5	29.7	29.7	30.8	29.7	29.7	29.8	30.2	30.1	30.5
≥ College (%)	25.3	26.3	11.0	34.3	34.8	14.4	35.9	36.4	13.1	32.0	32.7	12.3
**Annual Household Income**												
< $35,000 (%)	37.5	35.8	62.7	26.5	25.8	53.5	23.0	22.1	54.9	28.8	27.6	58.6
$35,000-$74,999 (%)	32.1	32.4	26.2	30.1	30.2	26.3	28.0	28.1	25.3	30.1	30.3	25.9
≥ $75,000 (%)	30.5	31.8	11.2	43.3	44.0	20.2	49.0	49.8	19.8	41.1	42.1	15.4
**Occupational Class**												
Professional/Management (%)	17.5	18.0	10.1	22.7	23.0	12.1	23.5	23.8	10.6	21.4	21.8	10.8
Support Services (%)	44.1	44.1	44.1	44.3	44.3	45.1	46.5	46.6	42.2	45.1	45.1	43.9
Laborers (%)	38.4	37.9	45.7	32.9	32.7	42.8	30.0	29.6	47.2	33.5	33.1	45.3
**Unemployed/not in the labor force** (%)	43.9	42.0	72.5	39.9	39.0	71.0	39.8	38.9	71.6	41.1	39.9	71.9
**Living in poverty (< 100% Federal Poverty Level)** (%)	15.5	14.3	33.9	9.9	9.4	25.3	8.1	7.6	26.4	11.0	10.3	29.8
**Other government assistance** (%)	17.9	16.5	40.3	10.8	10.2	32.4	8.1	7.6	31.3	12.1	11.3	36.1
**Region of residence**												
Northeast (%)	17.9	18.0	16.7	18.9	19.0	16.5	18.0	18.1	12.7	18.2	18.3	15.7
Midwest (%)	21.1	21.1	21.9	22.5	22.5	21.5	23.7	23.7	23.7	22.5	22.5	22.2
South (%)	36.4	36.2	38.7	35.6	35.6	38.4	37.8	37.8	41.3	36.7	36.6	39.3
West (%)	24.6	24.7	22.7	23.0	22.9	23.6	20.5	20.4	22.3	22.6	22.6	22.8
**Health Behaviors**												
**Sleep duration**												
< 6 h (%)	11.8	10.6	30.8	7.9	7.4	26.2	7.5	7.0	29.4	8.9	8.2	29.4
< 7 h (%)	24.5	24.4	26.7	22.6	22.5	26.4	20.4	20.3	25.5	22.4	22.2	26.2
7–9 h (%) (Recommended)	59.2	60.8	33.3	65.8	66.6	37.4	68.2	69.0	35.0	64.7	65.8	34.7
> 9 h (%)	4.5	4.2	9.3	3.6	3.5	10.0	3.9	3.8	10.1	4.0	3.8	9.6
**Trouble falling asleep** (≥ 3 times/week) (%)	25.5	22.9	68.0	18.7	17.4	65.1	16.7	15.6	63.7	20.1	18.4	66.4
**Trouble staying asleep** (≥ 3 times/week) (%)	32.4	30.1	69.8	26.3	25.1	69.9	24.5	23.5	66.9	27.5	25.9	69.2
**Woke up feeling unrested **(≥ 3 days/week) (%)	50.7	48.6	84.9	42.2	41.1	82.8	37.0	36.0	79.8	42.9	41.5	83.3
**Sleep medication use the past week **(≥ 3 times/week) (%)	11.5	9.9	35.3	8.9	8.3	32.9	9.1	8.4	37.8	9.8	8.8	35.2
**Smoking status**												
Never/ quit > 12 months prior^e^ (%)	80.4	81.6	61.4	84.7	85.3	65.2	85.6	86.2	62.0	83.7	84.5	62.7
Quit ≤ 12 months Ago (%)	1.5	1.4	2.7	1.3	1.3	2.0	1.2	1.2	2.6	1.3	1.3	2.5
Current (%)	18.1	17.0	35.9	14.0	13.4	32.7	13.2	12.7	35.4	15.0	14.2	34.7
**Leisure-time physical activity**												
Never/unable (%)	38.2	37.1	54.8	30.6	30.1	49.7	28.6	28.0	52.6	32.2	31.4	53.1
Does not meet PA guidelines (%)	19.3	19.3	19.4	19.1	19.0	22.2	18.2	18.2	18.3	18.9	18.8	19.8
Meets PA guidelines^b^^,e^ (%)	42.5	43.5	25.8	50.3	50.9	28.1	53.1	53.7	29.1	49.0	49.8	27.1
**Alcohol status**												
Never (%)	20.4	20.5	19.8	18.7	18.6	20.5	19.4	19.3	21.6	19.5	19.4	20.5
Former (%)	17.6	17.1	26.9	14.6	14.3	23.7	14.0	13.8	22.0	15.2	14.8	24.8
Current (%)	61.9	62.5	53.3	66.7	67.0	55.8	66.6	66.8	56.5	65.3	65.7	54.7
**Clinical Characteristics**												
**Body Mass Index (BMI)**												
Underweight (< 18.5 kg/m^2)^	1.6	1.5	2.8	1.6	1.5	3.7	1.6	1.6	2.8	1.6	1.6	3.1
Recommended body mass index (18.5- < 25 kg/m^2^)^e^ (%)	30.3	30.5	26.9	32.6	32.7	29.4	34.2	34.3	28.5	32.5	32.6	27.9
Overweight (25–29.9 kg/m^2^) (%)	34.0	34.4	27.6	36.0	36.2	29.7	36.0	36.1	30.3	35.4	35.6	28.7
Obese (≥ 30 kg/m^2^) (%)	34.1	33.6	42.6	29.8	29.6	37.1	28.2	28.0	38.4	30.5	30.2	40.4
**Dyslipidemia** (yes)^c,e^ (%)	50.9	49.8	64.1	48.6	48.2	58.6	50.2	49.7	64.3	49.8	49.1	62.7
**Hypertension** (yes)^e^ (%)	38.6	37.7	53.0	35.3	34.9	51.4	33.9	33.4	53.4	35.7	35.1	52.6
**Prediabetes/ diabetes (yes)**^e^** (%)**	20.9	20.2	31.5	17.4	17.0	30.9	15.4	15.1	28.7	17.6	17.1	30.1
**“Ideal” Cardiovascular Health (yes)**^d^ (%)	7.4	7.7	2.8	10.2	10.4	3.7	11.4	11.6	2.9	9.7	10.0	3.0
**Self-Rated Health Status**												
Excellent/Very Good/Good (%)	80.0	82.4	43.2	87.0	88.2	46.3	89.1	90.1	50.4	85.8	87.3	45.6
Fair/Poor (%)	20.0	17.6	56.8	13.0	11.8	53.7	10.9	9.9	49.6	14.2	12.7	54.4

All estimates are weighted for the survey’s complex sampling design. All estimates except for age are age-standardized to the U.S. 2010 population. Percentages may not sum to 100 due to missing values or rounding

*SE* standard error

^a^Kessler-6 psychological distress scale score ≥ 13

^b^Meets PA (physical activity) guidelines defined as ≥ 150 min/week of moderate intensity or ≥ 75 min/week of vigorous intensity or ≥ 150 min/week of moderate + vigorous intensity physical activity

^c^Dyslipidemia defined as high cholesterol in the 12 months prior to interview. Available for survey years 2011–2017

^d^“Ideal” cardiovascular health includes never smoking/quit > 12 months prior to interview, BMI 18.5- < 25 kg/m2, meeting physical activity guidelines, and no prior diagnosis of dyslipidemia, hypertension, or diabetes/prediabetes

^e^Indicator of “ideal” cardiovascular health

**Fig. 1:**
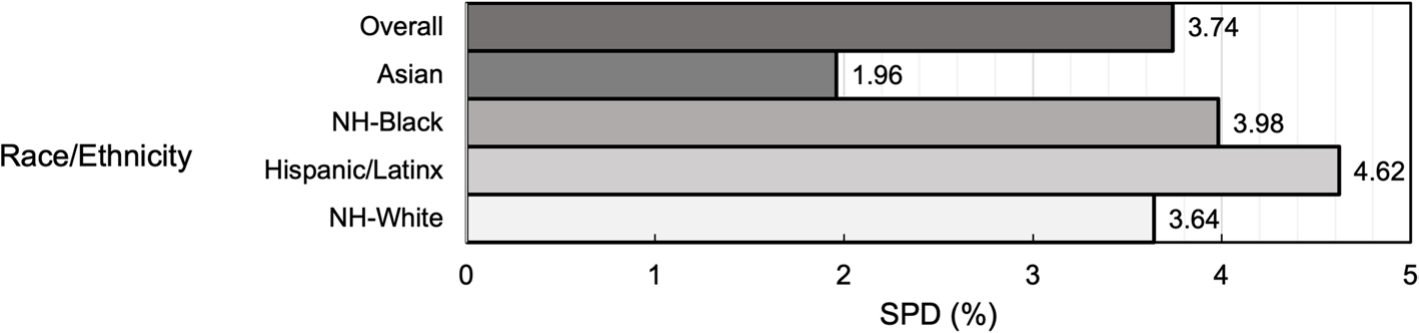
Prevalence of Serious Psychological Distress among the Overall Population and by Race/Ethnicity (*N* = 168,573). Percentage of participants overall and within racial/ethnic groups who have serious psychological distress. Serious psychological distress measured as score ≥ 13 based on the Kessler-6 scale

Overall, 3.7% of participants had SPD, while 2.0% of Asian, 4.0% of NH-Black, 4.6% of Hispanic/Latinx, and 3.6% of NH-White participants had SPD (Fig. 1). Among participants with SPD (3.7%), 60.3% were ≥ 50 years old, 61.7% were women, 2.9% were Asian, 11.5% were NH-Black, 17.5% were Hispanic/Latinx, 68.1% were NH-White, 84.5% had household incomes < $75,000, and 45.6% reported ≥ good health (Table 1). Further, 50.3% of participants strongly agreed there were people they could count on in their neighborhood, while 37.6% of those with SPD strongly agreed (Supplemental Table 1).

### Neighborhood social cohesion and serious psychological distress overall and by race/ethnicity

**Fig. 2:**
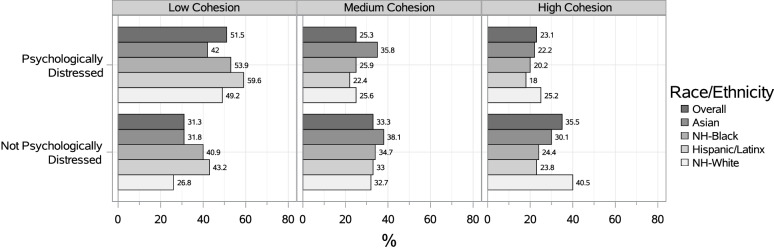
Prevalence of SPD by nSC Level, Overall and by Race/Ethnicity (*N* = 168,573). Percentage of participants (overall and by race/ethnicity) living in low, medium, and high levels of neighborhood social cohesion who are seriously psychologically distressed and not seriously psychologically distressed. Serious psychological distress measured as score ≥ 13 based on the Kessler-6 scale

Overall, the prevalence of SPD was highest among participants who reported living in neighborhoods with low social cohesion (51.5%) compared to medium (25.3%) and high (23.1%) (Fig. 2).

**Fig. 3:**
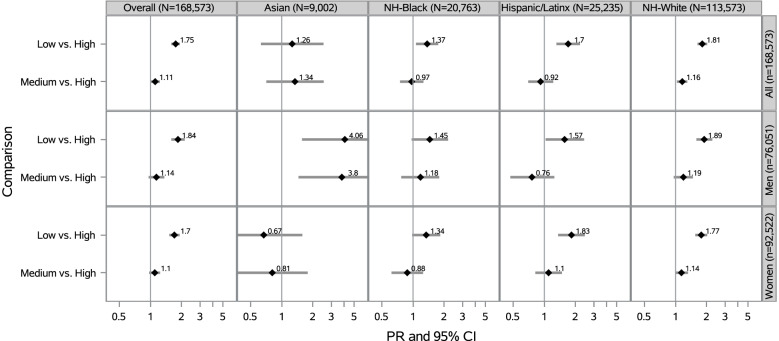
Prevalence Ratios of SPD by nSC, Overall and Stratified by Race/Ethnicity and Sex/Gender (N = 168,573). PR = Prevalence Ratio; CI = Confidence Interval; Adjusted for age (18–30, 31–49, ≥ 50 years), educational attainment (< high school, high school graduate, some college, ≥ college), annual household income (< $35,000, $35,000-$74,999, $75,000 +), occupational class (professional/management, support services, laborers), region of residence (Northeast, Midwest, South, West), alcohol consumption (never, former, current), “ideal” cardiovascular health (never smoking/quit > 12 months prior to interview, BMI 18.5- < 25 kg/m2, meeting physical activity guidelines, and no prior diagnosis of dyslipidemia, hypertension, or diabetes/prediabetes), marital/co-habiting status (married/living with partner or cohabitating, divorced/widowed/separated, single/no live-in partner), employment status (unemployed, employed), and self-rated health status (excellent/very good, good, fair/poor). All model additionally adjusted for sex/gender (woman, man). Overall models adjusted for race/ethnicity (NH-White, NH-Black, Hispanic/Latinx, and Asian). Note. All estimates are weighted for the survey’s complex sampling design. Interaction results between nSC*race/ethnicity were significant (*p*-value < 0.05) and interaction results between nSC*sex/gender were not statistically significant

Compared to participants who reported living in a neighborhood with high social cohesion, those who reported low nSC had a 75% higher prevalence of SPD (PR = 1.75 [95% CI: 1.59–1.92]) and those who reported medium nSC had an 11% higher prevalence of SPD (PR = 1.11 [95% CI: 1.00–1.23]), after adjustment (Fig. 3). Compared to their race/ethnicity counterparts who reported living in a neighborhood with high social cohesion, low nSC was associated with 26% higher prevalence of SPD among Asian participants (PR = 1.26 [95% CI: 0.63–2.53]), 37% higher prevalence of SPD among NH-Black participants (PR = 1.37 [95% CI: 1.07–1.75]), 70% higher prevalence of SPD among Hispanic/Latinx participants (PR = 1.70 [95% CI: 1.31–2.21]), and 81% higher prevalence of SPD among NH-White participants (PR = 1.81 [95% CI: 1.63–2.02]), after adjustment (Fig. 3).

**Fig. 4:**
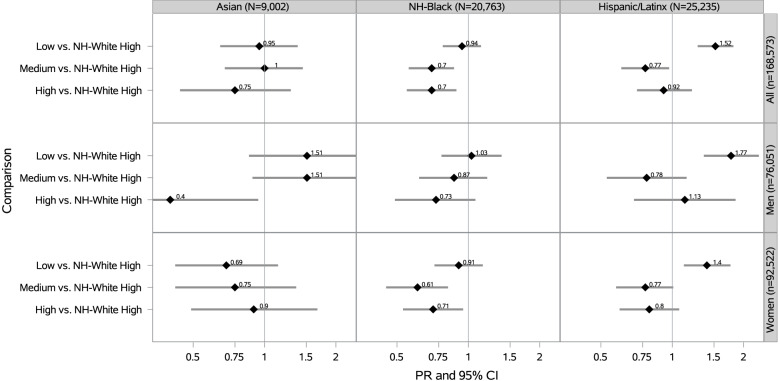
Prevalence Ratios of SPD by nSC: Racial Minoritized Participants vs. NH-Whites with High nSC (N = 168,573). PR = Prevalence Ratio; CI = Confidence Interval; Adjusted for age (18–30, 31–49, ≥ 50 years), educational attainment (< high school, high school graduate, some college, ≥ college), annual household income (< $35,000, $35,000-$74,999, $75,000 +), occupational class (professional/management, support services, laborers), region of residence (Northeast, Midwest, South, West), alcohol consumption (never, former, current), “ideal” cardiovascular health (never smoking/quit > 12 months prior to interview, BMI 18.5- < 25 kg/m2, meeting physical activity guidelines, and no prior diagnosis of dyslipidemia, hypertension, or diabetes/prediabetes), marital/co-habiting status (married/living with partner or cohabitating, divorced/widowed/separated, single/no live-in partner), employment status (unemployed, employed), and self-rated health status (excellent/very good, good, fair/poor). All model additionally adjusted for sex/gender (woman, man). Note. All estimates are weighted for the survey’s complex sampling design

We also investigated nSC and SPD among minoritized racial/ethnic participants compared to NH-White participants. Compared to NH-White participants living in neighborhoods with high social cohesion, NH-Black participants in medium and high nSC had a 30% lower prevalence of SPD (PR_medium_ = 0.70 [95% CI: 0.56–0.87]; PR_high_ = 0.70 [95% CI: 0.55–0.89]), after adjustment (Fig. 4). Compared to NH-White participants living in neighborhoods with high social cohesion, Hispanic/Latinx participants living in neighborhoods with low social cohesion had a 52% higher prevalence of SPD (PR = 1.52 [95% CI: 1.28–1.81]) (Fig. 4).

### Neighborhood social cohesion and serious psychological distress within racial/ethnic groups by sex/gender

Among Asian participants, low vs. high nSC was associated with a 4 times the prevalence of SPD among Asian men (PR = 4.06 [95% CI: 1.57–10.50]) and a 33% lower prevalence of SPD among Asian women (PR = 0.67 [95% CI: 0.29–1.58]) (Fig. 3). There were no other public health relevant modifications of the nSC-SPD association within racial/ethnic groups by sex/gender [52].

### Neighborhood social cohesion and serious psychological distress within racial/ethnic groups by self-rated health status

**Fig. 5:**
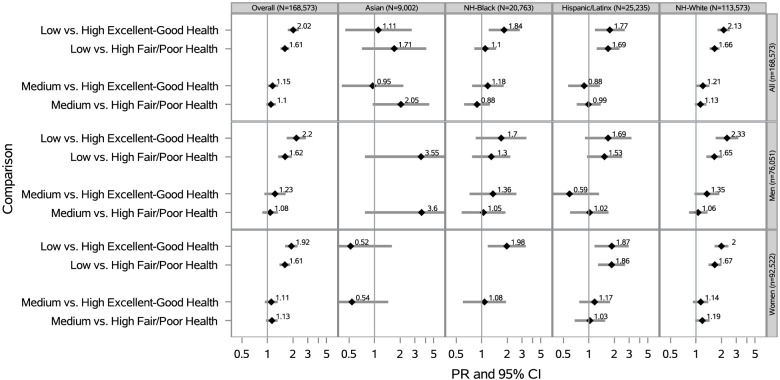
Prevalence Ratios of SPD by nSC, Overall, Stratified by Race/Ethnicity, Sex/Gender, Self-Rated Health Status (N = 168,573). PR = Prevalence Ratio; CI = Confidence Interval;Adjusted for age (18–30, 31–49, ≥ 50 years), educational attainment (< high school, high school graduate, some college, ≥ college), annual household income (< $35,000, $35,000-$74,999, $75,000 +), occupational class (professional/management, support services, laborers), region of residence (Northeast, Midwest, South, West), alcohol consumption (never, former, current), “ideal” cardiovascular health (never smoking/quit > 12 months prior to interview, BMI 18.5- < 25 kg/m2, meeting physical activity guidelines, and no prior diagnosis of dyslipidemia, hypertension, or diabetes/prediabetes), marital/co-habiting status (married/living with partner or cohabitating, divorced/widowed/separated, single/no live-in partner), and employment status (unemployed, employed).All model additionally adjusted for sex/gender (woman, man) Overall models adjusted for race/ethnicity. Note. All estimates are weighted for the survey’s complex sampling design. Interaction results between nSC*self-rated health status were significant (p-value < 0.05). Blanks indicate data that was not estimable

Overall, low vs. high nSC was associated with a 2 times the prevalence of SPD for participants in at least good health (PR = 2.02 [95% CI: 1.74–2.35]) and a 61% higher prevalence of SPD for those in fair/poor health (PR = 1.61 [95% CI: 1.43–1.81]), after adjustment (Fig. 5). Among NH-White participants, low vs. high nSC was associated with 2.13 times the prevalence of SPD for those in at least good health (PR = 2.13 [95% CI: 1.80–2.51]) and 1.66 times the prevalence of SPD for those in fair/poor health (PR = 1.66 [95% CI: 1.45–1.90]), after adjustment (Fig. 5). There were no other public health relevant modifications of the nSC-SPD association within racial/ethnic groups by self-rated health status.

### Neighborhood social cohesion and serious psychological distress within racial/ethnic groups by age

**Fig. 6:**
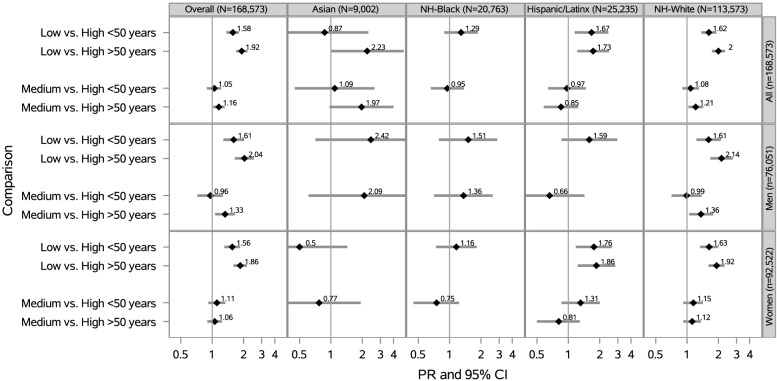
Prevalence Ratios of SPD by nSC, Overall and Stratified by Race/Ethnicity, Sex/Gender, and Age (N = 168,573). PR = Prevalence Ratio; CI = Confidence Interval; Adjusted for educational attainment (< high school, high school graduate, some college, ≥ college), annual household income (< $35,000, $35,000-$74,999, $75,000 +), occupational class (professional/management, support services, laborers), region of residence (Northeast, Midwest, South, West), alcohol consumption (never, former, current), “ideal” cardiovascular health (never smoking/quit > 12 months prior to interview, BMI 18.5- < 25 kg/m2, meeting physical activity guidelines, and no prior diagnosis of dyslipidemia, hypertension, or diabetes/prediabetes), marital/co-habiting status (married/living with partner or cohabitating, divorced/widowed/separated, single/no live-in partner), employment status (unemployed, employed), and self-rated health status (excellent/very good, good, fair/poor). All model additionally adjusted for sex/gender (woman, man). Overall models adjusted for race/ethnicity. Note. All estimates are weighted for the survey’s complex sampling design. Interaction results between nSC*age were not statistically significant (p-value < 0.10). Blanks indicate data that was not estimable

Overall, low vs. high nSC was associated with a 92% higher prevalence of SPD for participants ≥ 50 years old (PR = 1.92 [95% CI: 1.70–2.18]) and 58% higher prevalence of SPD for participants < 50 years old (PR = 1.58 [95% CI: 1.37–1.81]), after adjustment (Fig. 6). There were no public health relevant modifications of the nSC-SPD association within racial/ethnic groups by age.

### Neighborhood social cohesion and serious psychological distress within racial/ethnic groups by annual household income

**Fig. 7:**
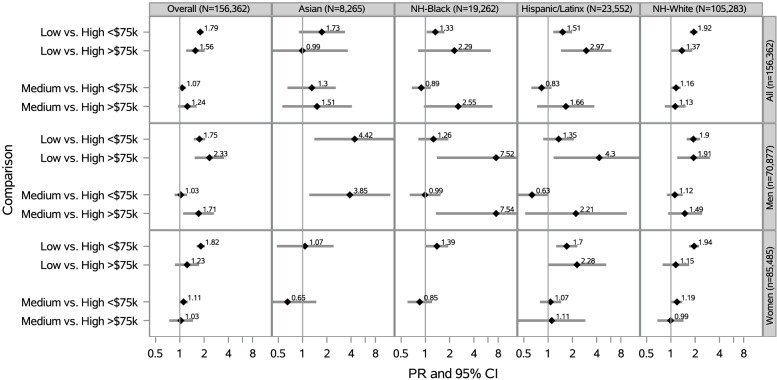
Prevalence Ratios of SPD by nSC, Overall, Stratified by Race/Ethnicity, Sex/Gender, Annual Household Income (*N* = 156,362)^a^. ^a^12211 participants excluded for missing income data. PR = Prevalence Ratio; CI = Confidence Interval; Adjusted for educational attainment (< high school, high school graduate, some college, ≥ college), age (18–30, 31–49, ≥ 50 years), occupational class (professional/management, support services, laborers), region of residence (Northeast, Midwest, South, West), alcohol consumption (never, former, current), “ideal” cardiovascular health (never smoking/quit > 12 months prior to interview, BMI 18.5- < 25 kg/m2, meeting physical activity guidelines, and no prior diagnosis of dyslipidemia, hypertension, or diabetes/prediabetes), marital/co-habiting status (married/living with partner or cohabitating, divorced/widowed/separated, single/no live-in partner), employment status (unemployed, employed), and self-rated health status (excellent/very good, good, fair/poor).All model additionally adjusted for sex/gender (woman, man). Overall models adjusted for race/ethnicity. Note. All estimates are weighted for the survey’s complex sampling design. Interaction results between nSC*annual household income were not statistically significant. Blanks indicate data that was not estimable

Among Hispanic/Latinx participants overall, low vs. high nSC was associated with a 1.51 times the prevalence of SPD for those with household incomes < $75,000 (PR = 1.51 [95% CI: 1.16–1.98]) and 2.97 times the prevalence of SPD for those with incomes ≥ $75,000 (PR = 2.97 [95% CI: 1.45–6.08]), after adjustment (Fig. 7). Among NH-White participants, low vs. high nSC was associated with 92% higher prevalence of SPD for those with household incomes < $75,000 (PR = 1.92 [95% CI: 1.71–2.15]) and 37% higher prevalence of SPD for those with incomes ≥ $75,000 (PR = 1.37 [95% CI: 1.02–1.84]), after adjustment.

### Sensitivity analyses

Results remained robust after considering both length of residence as a potential confounder of the nSC-SPD relationship and psychological vs. serious psychological distress as an additional outcome category (Supplemental Tables 2 and 3).

## Discussion

Among a large, nationally representative study in the US, we found levels of nSC were evenly distributed in the study population and the prevalence of SPD was approximately 4%, which is consistent with prior literature [4]. However, we also found that NH-Black, Hispanic/Latinx, and participants with lower household incomes were disproportionately represented among those who reported low nSC while Hispanic/Latinx participants, women, and lower income participants were overrepresented among those with SPD. Overall, participants who perceived their neighborhoods as having low vs. high social cohesion were more likely to report SPD, which was consistent with our hypotheses. Our hypotheses were also supported by the findings that the association between low vs. high nSC and SPD was stronger among participants who reported at least good compared to fair/poor health and in older compared to younger participants. However, our overall findings by race/ethnicity, sex/gender, and annual household income did not support our hypotheses. We observed that the association between low vs. high nSC and SPD was stronger in NH-White participants than in NH-Black, Hispanic/Latinx, and Asian participants. However, SPD was most prevalent among Hispanic/Latinx, NH-Black and NH-White, then Asian participants, which is relevant to public health burden. While investigating potential modifiers, we observed variations in the association between low vs. high nSC and SPD by sex/gender among Asian participants only, where the association between low nSC and higher prevalence of SPD was stronger among Asian men compared to Asian women. Our results stratified by household income varied, where the association between low nSC and higher SPD was stronger among those with household incomes ≥ $75,000 vs. < $75,000 in Hispanic/Latinx participants and we found the opposite among NH-White participants. We found effect modification by race/ethnicity, self-rated health status, and age in the overall population. We also found effect modification by sex/gender among Asian participants and by annual household income among Hispanic/Latinx and NH-White participants. Finally, our sensitivity results remained robust when also considering clinically relevant psychological distress (which is indicated by a K6 score of 5–12 compared to a score of ≥ 13 for SPD) below the threshold for SPD.

Our finding that low vs. high nSC was associated with higher SPD was consistent with previous studies examining SPD or psychological distress in general [6, 18, 33, 39, 40]. For example, one study found that higher levels of nSC were associated with lower psychological distress in the United Kingdom [39]. Similarly, another study found that living in higher nSC was related to lower psychological distress among Hispanic/Latinx and NH-residents of Phoenix, Arizona [19]. Since we used a large, multi-ethnic diverse sample, our results expand upon the growing body of literature regarding associations between nSC and mental health.

Contrary to our hypothesis, the strongest associations between low vs. high nSC and SPD were observed among NH-Whites compared to other racial/ethnic groups. There are several potential explanations for our results. Because neighborhoods tend to be comprised primarily of one racial/ethnic group due to racial residential segregation [53], racial/ethnic enclaves among minoritized groups (e.g., Hispanic/Latinxs) may, despite levels of nSC, buffer against SPD by fostering strong social ties, increasing access to health-promoting services, and mitigating adverse health effects of racial/ethnic discrimination [36, 53–55]. However, this may not fully explain our findings as prior work demonstrates that even racial/ethnic enclaves do not compensate for general neighborhood deprivation regardless of nSC level [54, 56]. Minoritized racial/ethnic groups, who typically live in resource-deprived neighborhoods created by structural racism, experience social disadvantages that may compete with the potential impact of low nSC. NH-Whites, on the other hand, are more likely to live in resource-rich neighborhoods with fewer stressors or social disadvantages. Therefore, they may experience positive impacts on health from high nSC while lower perceptions of nSC can have more negative impacts on health than in other groups with many competing disadvantages. Further, the prevalence of SPD was lowest among Asian participants, which may be explained by factors such as stigma and relative emphases on collectivism vs. individualism [57, 58]. Another potential explanation may involve the measurement of nSC. Although this scale has been validated in the general population [59], it has not been validated, to our knowledge, across all the racial/ethnic groups included this is study. Therefore, this measure may have limited validity to capture components of nSC relevant to different racial/ethnic groups [60, 61].

When comparing minoritized racial/ethnic participants living in low nSC to NH-White participants living in high nSC, we found that Hispanic/Latinx participants had higher SPD. These findings are similar to another study, which found a stronger association between higher nSC and lower psychological distress among Hispanics/Latinxs than in NH participants [19]. Our results extend the prior literature by demonstrating the same association among a large, representative Hispanic/Latinx sample. We also found that NH-Black participants living in medium and high nSC experienced *lower* SPD compared to NH-White participants living in high nSC. Because these results are across racial/ethnic groups, they may highlight differences by race/ethnicity, where it is possible that nSC may have a stronger impact on mental health in minoritized populations where nSC and social support may protect against SPD [20]. These results may also be explained by the stigma surrounding poor mental health among minoritized racial/ethnic groups, ethnic enclaves, differential perception and reporting of SPD by race/ethnicity, and/or the measurement of nSC [54, 55, 57, 62, 63].

Our finding that there was no effect modification for low vs. high nSC and SPD by sex/gender in the overall population did not support our hypothesis. Prior literature has demonstrated that women are more influenced by social environmental factors, such as safety and social support [24–26], and that women have higher SPD than men[4]. Few studies, however, examined the potential modifying role that sex/gender has on the association between nSC and SPD [20]. Since women often engage in their social neighborhood environment more than men, it is possible that women who live in neighborhoods with low nSC seek alternative avenues of social engagement that may protect against SPD [64]. Among Asian participants, the association between low nSC and higher SPD was much stronger among Asian men compared to Asian women, although the CI for Asian men was relatively wide, and there was slight overlap with Asian women. Nevertheless, this finding appears to have public health relevance. This finding is interesting since, on average, the influence of nSC is typically stronger for women than men and previous studies have shown that SPD and other mental disorders are generally more prevalent in Asian women than Asian men [65]. This underscores the importance of explicitly investigating the complexities and nuances across intersectional identities since relationships can differ in ways that have important implications for interventions. Therefore, additional research is needed to further understand how neighborhood contexts can affect health across various sociodemographic groups, especially at the intersection of race/ethnicity and sex/gender.

In the overall population and among NH-White participants, we found that the association between low nSC and higher SPD was stronger among those in at least good health compared to those in fair/poor health. Although there was some CI overlap, these results suggest effect modification by self-rated health status. While it may not be intuitive for low vs. high nSC to be associated with higher SPD among those with at least good health vs. fair/poor health, we hypothesized this for several reasons. Since the average age of our sample was 47 ± 0.1 years, we are most likely capturing middle-aged adults at the peak of their life who may experience at least good health while concurrently experiencing heightened stressors (e.g., job-related stress) [66]. So, it is possible that the neighborhood environment may have an impact on SPD regardless of general self-rated health status. Further, poor self-rated health is consistently associated with psychological distress [67], so it is possible that poor self-rated health status masks the association between nSC and SPD where we may have only captured the association between nSC and SPD among participants with at least good health. A similar relationship between self-rated health status and SPD has been observed in a previous study, which also used NHIS data from 2004 to 2017 [30]. Finally, our small sample size for participants in poor health (n = 23,937) compared to those with at least good health (n = 144,636) may have decreased our power to detect differences.

Our findings that the association between low nSC and higher SPD was stronger among participants ≥ 50 years old compared to those < 50 years old supported our hypothesis. Although there was slight CI overlap in our results, our findings are consistent with previous studies that examined the relationship between nSC and psychological distress in older adults [33, 38]. For example, one study found that higher vs. lower nSC was associated with lower psychological distress among US adults > 50 years old [33]. Since some data by race/ethnicity-age-sex/gender was not estimable, future studies should consider multiple social categories (e.g., race/ethnicity-sex/gender-age) to understand the relationship between nSC and SPD.

Among NH-White participants, we found that the association between low nSC and higher SPD was stronger among those with annual household incomes < $75,000 compared to those with annual household incomes ≥ $75,000, which supported our hypothesis. Although there was slight CI overlap, this finding suggests that the neighborhood social environment is important to mental health among those with lower incomes. These findings are similar to a study conducted in the Netherlands, which found that low vs. high nSC was associated with higher risk of psychological distress among those with financial difficulties compared to those without financial difficulties [18]. Our findings among Hispanic/Latinx participants did not corroborate our hypothesis, as we found that the association between low nSC and higher SPD was stronger for Hispanic/Latinx participants with annual household incomes ≥ $75,000 compared to those with incomes < $75,000. These findings may be explained by racial/ethnic enclaves and differential perception of nSC by race/ethnicity [54, 63]. Moreover, although affluent individuals from racially/ethnically minoritized groups are less likely to live in affluent neighborhoods than NH-Whites due to racial and economic residential segregation [68], Hispanic/Latinx individuals with high incomes may be more likely than low-income Hispanic/Latinxs to live in affluent, predominantly NH-White neighborhoods [53, 69]. Therefore, it is possible that we observed a higher prevalence of SPD among higher income Hispanic/Latinxs because this group may feel like an outsider or discriminated against in affluent communities and consequently perceive less nSC. Given the lack of available data in NHIS to test this idea, this should be investigated in future research. Despite some confidence interval overlap, our results suggest effect modification by household income in Hispanic/Latinx and NH-White participants. Further, these results reveal another example of how the neighborhood environment can differentially impact health when various social identities intersect (i.e., race/ethnicity and household income).

It is hypothesized that nSC influences SPD through sociological, environmental, and biological pathways. Living in a neighborhood with low levels of social cohesion may reinforce unhealthy behaviors and increase feelings of perceived vigilance and social exclusion, which may increase SPD [10, 11, 19, 51, 70, 71]. Reinforcing unhealthy behaviors like alcohol consumption, smoking, physical inactivity, poor sleep, and poor diet may manifest to SPD [14, 15, 17, 51, 71–74]. For example, long-term alcohol consumption and cigarette smoking increase the activation of corticotropin-releasing-hormone in the hypothalamus through excitatory neurotransmission and increase nicotine levels and subsequently release adrenocorticotropic hormone from the anterior pituitary [14, 17]. Increased vigilance and feelings of exclusion may also contribute to the practice of unhealthy behaviors and may also directly increase activation of the limbic system, including the amygdala, insula, and anterior cingulate cortex [11, 13, 16]. Each of these processes directly contributes to the activation of the HPA axis and thus, the release of cortisol [13], where excessive, prolonged, and/or unmanaged experiences of stress lead to SPD [75]. Conversely, living in a neighborhood with high levels of social cohesion may reinforce healthy behaviors, such as sleeping at an earlier time, and increase feelings of perceived safety and social support [18, 19]. Giving and receiving social support may decrease the negative effects of social stressors (e.g., financial difficulties), prompt positive psychological responses (e.g., sense of belonging), and increase motivation for self-preservation [76], which can alleviate psychosocial stress and thus contribute to better mental health outcomes [20].

Our study has several limitations. Because of the cross-sectional design of the study, we are unable to infer on causality between nSC and SPD. The use of subjective, self-reported data for both nSC and SPD variables may have resulted in measurement bias [77]. Specifically, perceptions of nSC may vary by neighbors and stigma surrounding mental illness may influence how participants respond to survey items [78]. However, studies show that individuals’ perception of their neighborhood tends to be more strongly associated with poor health outcomes than objective neighborhood measures and that the K6 scale is a valid and reliable measure to capture SPD across racial/ethnic groups in the US [46, 79]. Further, although the modified four statements used to measure nSC by the NHIS have been used in prior studies [44, 45], to our knowledge, it has not been validated against the original 5-item survey. Since individuals with a previously diagnosed mental illness may perceive their neighborhoods to be less socially cohesive than those without a prior diagnosis, reverse causation is possible and may overestimate the observed associations [80]. Additionally, the dichotomization of age using cut-off ≥ 50 years old may not allow for an in-depth analysis of nSC and SPD by age; however, previous work has shown that the prevalence of SPD is highest among those averaging 50 years old [4]. Another limitation is the exclusion of indigenous groups including Native Americans, American Indians, and Alaskan Natives due to a small sample size. Finally, the binary sex/gender variable (i.e., man/woman) used by the NHIS did not account for people who do not fall within the gender binary (e.g., nonbinary individuals).

Despite these limitations, this study has noteworthy strengths. Our study used a large, nationally representative sample that allows our results to be generalized to the Asian, NH-Black, Hispanic/Latinx, and NH-White US adult population. Our sample consisted of six years of NHIS data, which markedly increased our sample size and decreased the likelihood of non-representative results stemming from data collected in just a single calendar year. Furthermore, this large sample size allowed us to robustly examine the novel interactions of race/ethnicity, sex/gender, self-rated health status, age, and annual household income, separately and combined, between nSC and SPD. We were also able to conduct analyses regarding intersectionality, which can begin to reveal the informative complexities as well as nuances regarding how neighborhood context can affect health across various groups or social identities. Finally, our results remained robust after including length of residence as an additional confounder in a sensitivity analysis as well as including psychological distress as an additional category of the outcome.

## Conclusion

This study adds to the growing body of research investigating nSC and SPD by demonstrating an association between low vs. high nSC and SPD among the overall population, NH-White individuals, Asian men, those ≥ 50 years old, and Hispanic/Latinx individuals with household incomes ≥ $75,000. Our findings may inform future community interventions that target the neighborhood environment as well as stress management interventions that focus on specific sociodemographic groups, including at the intersections of social identities. Improving nSC may potentially reduce SPD and overall health in a practical and effective way. For example, investing in economically disadvantaged communities by establishing community coalitions and providing more opportunities for social engagement through, for instance, improved infrastructure and increased access to green space may improve nSC [81, 82].

In conclusion, our nationally representative study suggests that the impact of neighborhood conditions (either physical or social) on mental health may be differentially important for certain intersecting sociodemographic groups. These findings underscore the importance of examining nSC and other upstream determinants of SPD through an intersectional lens in the overall population, which can inform future neighborhood-level interventions and policies designed to improve mental health and address health disparities.

## Supplementary Information

**Additional file 1: Supplemental figure 1.** Composition of Analytic Sample.

**Additional file 2: Supplemental Table 1.** Age-Standardized Sociodemographic Characteristics across components of the Neighborhood Social Cohesion Scale (*N*=168,573).

**Additional file 3: Supplemental Table 2.** Prevelence Ratios of PD and SPD by nSC, Overall and Stratified by Race/Ethnicity, Sex/Gender (*N*=168,573).

**Additional file 4: Supplemental Table 3.** Prevelence Ratios of PD and SPD: Racial Minoritized Participants vs. NH-Whites with High nSC (*N*=168,573).

## Data Availability

The datasets generated and/or analyzed for this study are publicly available on the Integrated Health Interview Series website (https://nhis.ipums.org/nhis/).

## Abbreviations

BMI: Body Mass Index
CI: Confidence Interval
NH: Non-Hispanic
nSC: Neighborhood Social Cohesion
PR: Prevalence Ratio
SPD: Serious Psychological Distress
US: United States

## References

[1] Tsai CC, Chuang SY, Hsieh IC, Ho LH, Chu PH, Jeng C (2019). The association between psychological distress and angina pectoris: A population-based study. PLoS ONE.

[2] van Dijk I, Lucassen P, van Weel C, Speckens AEM (2017). A cross-sectional examination of psychological distress, positive mental health and their predictors in medical students in their clinical clerkships. BMC Med Educ.

[3] Arvidsdotter T, Marklund B, Kylen S, Taft C, Ekman I (2016). Understanding persons with psychological distress in primary health care. Scand J Caring Sci.

[4] Weissman J, Pratt LA, Miller EA, Parker JD (2015). Serious Psychological Distress Among Adults: United States, 2009–2013.

[5] Forman-Hoffman , , , ,  VL, Muhuri PK, Novak SP, Pemberton MR, Ault KL, Mannix D (2014). Psychological Distress and Mortality among Adults in the U.S. Household Population. The CBHSQ Report.

[6] Holmes LM, Marcelli EA (2020). Neighborhood Social Cohesion and Serious Psychological Distress Among Brazilian Immigrants in Boston. Community Ment Health J.

[7] Florez KR, Ghosh-Dastidar MB, Beckman R, de la Haye K, Duru OK, Abraido-Lanza AF (2016). The Power of Place: Social Network Characteristics, Perceived Neighborhood Features, and Psychological Distress Among African Americans in the Historic Hill District in Pittsburgh. Pennsylvania Am J Community Psychol.

[8] Lim S, Meausoone V, Norman C, Quinlan C, Driver CR (2017). Neighborhood contributions to psychological distress among Latino New York City adults. Ethn Health.

[9] Brisson D (2014). Neighborhood social cohesion.

[10] Jacobsen JC, Maytal G, Stern TA (2007). Demoralization in medical practice. Prim Care Companion J Clin Psychiatry.

[11] Morese R, Lamm C, Bosco FM, Valentini MC, Silani G (2019). Social support modulates the neural correlates underlying social exclusion. Soc Cogn Affect Neurosci.

[12] Cutrona CE, Russell DW, Hessling RM, Brown PA, Murry V (2000). Direct and moderating effects of community context on the psychological well-being of African American women. J Pers Soc Psychol.

[13] Eisenberger NI, Taylor SE, Gable SL, Hilmert CJ, Lieberman MD (2007). Neural pathways link social support to attenuated neuroendocrine stress responses. Neuroimage.

[14] Mendelson JH, Sholar MB, Goletiani N, Siegel AJ, Mello NK (2005). Effects of low- and high-nicotine cigarette smoking on mood states and the HPA axis in men. Neuropsychopharmacol.

[15] Stephens MA, Wand G (2012). Stress and the HPA axis: role of glucocorticoids in alcohol dependence. Alcohol Res.

[16] Peters AT, Van Meter A, Pruitt PJ, Briceno EM, Ryan KA, Hagan M (2016). Acute cortisol reactivity attenuates engagement of fronto-parietal and striatal regions during emotion processing in negative mood disorders. Psychoneuroendocrinol.

[17] Valenzuela CF (1997). Alcohol and neurotransmitter interactions. Alcohol Health Res World.

[18] Erdem O, Van Lenthe FJ, Prins RG, Voorham TA, Burdorf A (2016). Socioeconomic Inequalities in Psychological Distress among Urban Adults: The Moderating Role of Neighborhood Social Cohesion. PLoS ONE.

[19] Rios R, Aiken LS, Zautra AJ (2012). Neighborhood contexts and the mediating role of neighborhood social cohesion on health and psychological distress among Hispanic and non-Hispanic residents. Ann Behav Med.

[20] Henderson H, Child S, Moore S, Moore JB, Kaczynski AT (2016). The Influence of Neighborhood Aesthetics, Safety, and Social Cohesion on Perceived Stress in Disadvantaged Communities. Am J Community Psychol.

[21] Bailey ZD, Krieger N, Agenor A, Graves J, Linos N, Bassett MT (2017). Structural racism and health inequities in the USA: evidence and interventions. Lancet.

[22] Williams DR, Collins C (2001). Racial residential segregation: a fundamental cause of racial disparities in health. Public Health Rep.

[23] Stewart D-L (2013). Racially Minoritized Students at U.S. Four-Year Institutions. J Negro Educ.

[24] Guilcher SJT, Kaufman-Shriqui V, Hwang J, O'Campo P, Matheson FI, Glazier RH (2017). The association between social cohesion in the neighborhood and body mass index (BMI): An examination of gendered differences among urban-dwelling Canadians. Prev Med.

[25] Stafford M, Cummins S, Macintyre S, Ellaway A, Marmot M (2005). Gender differences in the associations between health and neighborhood environment. Soc Sci Med.

[26] Cutrona CE, Wallace G, Wesner KA (2006). Neighborhood Characteristics and Depression: An Examination of Stress Processes. Curr Dir Psychol Sci.

[27] Ou JY, Peters JL, Levy JI, Bongiovanni R, Rossini A, Scammell MK (2018). Self-rated health and its association with perceived environmental hazards, the social environment, and cultural stressors in an environmental justice population. BMC Public Health.

[28] Yang H, Deng Q, Geng Q, Tang Y, Ma J, Ye W (2021). Association of self-rated health with chronic disease, mental health symptom and social relationship in older people. Sci Rep.

[29] Gebauer S, Schootman M, Xian H, Xaverius P (2020). Neighborhood built and social environment and meeting physical activity recommendations among mid to older adults with joint pain. Prev Med Rep.

[30] Goldstein SJ, Gaston SA, McGrath JA, Jackson CL (2020). Sleep Health and Serious Psychological Distress: A Nationally Representative Study of the United States among White, Black, and Hispanic/Latinx Adults. Nat Sci Sleep.

[31] Brim B, Fromhold S, Blaney S (2021). Older Adults' Self-Reported Barriers to Aging in Place. J Appl Gerontol.

[32] Cagney KA, Browning CR, Wen M (2005). Racial disparities in self-rated health at older ages: What difference does the neighborhood make?. J Gerontol B-Psychol.

[33] Kim ES, Chen Y, Kawachi I, VanderWeele TJ (2020). Perceived neighborhood social cohesion and subsequent health and well-being in older adults: An outcome-wide longitudinal approach. Health Place.

[34] Owens A (2019). Building Inequality: Housing Segregation and Income Segregation. Sociological Science.

[35] Cubbin C, Pedregon V, Egerter S, Braveman P. Where we live matters for our health: neighborhoods and health. Princeton: Robert Wood Johnson Foundation; 2008.

[36] Hong S, Zhang W, Walton E (2014). Neighborhoods and mental health: exploring ethnic density, poverty, and social cohesion among Asian Americans and Latinos. Soc Sci Med.

[37] Jackson CL, Redline S, Emmons KM. Sleep as a potential fundamental contributor to disparities in cardiovascular health. San Mateo: Annual Review of Public Health: Annual Reviews Inc.; 2015. p. 417–40. 10.1146/annurev-publhealth-031914-122838 PMC4736723 25785893

[38] Choi YJ, Matz-Costa C (2018). Perceived Neighborhood Safety, Social Cohesion, and Psychological Health of Older Adults. Gerontologist.

[39] Papachristou E, Flouri E, Kokosi T, Francesconi M (2019). Main and interactive effects of inflammation and perceived neighbourhood cohesion on psychological distress: results from a population-based study in the UK. Qual Life Res.

[40] Zhang W, Liu S, Zhang K, Wu B (2020). Neighborhood Social Cohesion, Resilience, and Psychological Well-Being Among Chinese Older Adults in Hawai'i. Gerontologist.

[41] IPUMS Health Surveys: National Health Interview Survey, Version 6.2. 2017. Available from: https://nhis.ipums.org/nhis/.

[42] 2015 National Health Interview Survey Description. National Center for Health Statistics, Centers for Disease Control and Prevention. Hyattsville: U.S. Department of Health and Human Services; 2016.

[43] Sampson RJ, Raudenbush SW, Earls F (1997). Neighborhoods and violent crime: a multilevel study of collective efficacy. Science.

[44] Alhasan DM, Gaston SA, Jackson WB, Williams PC, Kawachi I, Jackson CL (2020). Neighborhood Social Cohesion and Sleep Health by Age, Sex/Gender, and Race/Ethnicity in the United States. Int J Environ Res Public Health.

[45] Young MC, Gerber MW, Ash T, Horan CM, Taveras EM (2018). Neighborhood social cohesion and sleep outcomes in the Native Hawaiian and Pacific Islander National Health Interview Survey. Sleep..

[46] Kessler RC, Barker PR, Colpe LJ, Epstein JF, Gfroerer JC, Hiripi E (2003). Screening for serious mental illness in the general population. Arch Gen Psychiat.

[47] Piercy KL, Troiano RP, Ballard RM, Carlson SA, Fulton JE, Galuska DA (2018). The Physical Activity Guidelines for Americans. JAMA.

[48] Lloyd-Jones DM, Hong Y, Labarthe D, Mozaffarian D, Appel LJ, Van Horn L (2010). Defining and setting national goals for cardiovascular health promotion and disease reduction: the American Heart Association's strategic Impact Goal through 2020 and beyond. Circulation.

[49] Rodrigues DE, Cesar CC, Xavier CC, Caiaffa WT, Proietti FA (2015). The place where you live and self-rated health in a large urban area. Cad Saude Publica.

[50] Barros AJ, Hirakata VN (2003). Alternatives for logistic regression in cross-sectional studies: an empirical comparison of models that directly estimate the prevalence ratio. BMC Med Res Methodol.

[51] Pabayo R, Grinshteyn E, Avila O, Azrael D, Molnar BE (2020). Relation between neighborhood socio-economic characteristics and social cohesion, social control, and collective efficacy: Findings from the Boston Neighborhood Study. Ssm-Popul Hlth.

[52] Karpen SC (2017). P Value Problems. Am J Pharm Educ.

[53] Logan JR (2013). The Persistence of Segregation in the 21(st) Century Metropolis. City Community..

[54] Li K, Wen M, Henry KA (2017). Ethnic density, immigrant enclaves, and Latino health risks: A propensity score matching approach. Soc Sci Med.

[55] Williams AD, Messer LC, Kanner J, Ha S, Grantz KL, Mendola P (2020). Ethnic Enclaves and Pregnancy and Behavior Outcomes Among Asian/Pacific Islanders in the USA. J Racial Ethn Health Disparities.

[56] Osypuk TL, Diez Roux AV, Hadley C, Kandula NR (2009). Are immigrant enclaves healthy places to live? The Multi-ethnic Study of Atherosclerosis. Soc Sci Med.

[57] Budhwani H, De P (2019). Perceived Stigma in Health Care Settings and the Physical and Mental Health of People of Color in the United States. Health Equity.

[58] Papadopoulos C, Foster J, Caldwell K (2013). 'Individualism-collectivism' as an explanatory device for mental illness stigma. Community Ment Health J.

[59] Cagney KA, Glass TA, Skarupski KA, Barnes LL, Schwartz BS, Mendes de Leon CF (2009). Neighborhood-level cohesion and disorder: measurement and validation in two older adult urban populations. J Gerontol B Psychol Sci Soc Sci.

[60] Karasz A, Gany F, Escobar J, Flores C, Prasad L, Inman A (2019). Mental Health and Stress Among South Asians. J Immigr Minor Health.

[61] Gilbert KL, Ransome Y, Dean LT, DeCaille J, Kawachi I (2022). Social Capital, Black Social Mobility, and Health Disparities. Annu Rev Public Health.

[62] Conner KO, Copeland VC, Grote NK, Koeske G, Rosen D, Reynolds CF (2010). Mental health treatment seeking among older adults with depression: the impact of stigma and race. Am J Geriatr Psychiatry.

[63] Villatoro AP, Mays VM, Ponce NA, Aneshensel CS (2018). Perceived Need for Mental Health Care: The Intersection of Race, Ethnicity, Gender, and Socioeconomic Status. Soc Ment Health.

[64] Urzua CB, Ruiz M, Pajak A, Kozela M (2018). The Prospective Relationship Between Social Cohesion and Depressive Symptoms Among Older Adults From Central and Eastern Europe. J Epidemiol Community Health.

[65] Cheng AW, Lee CS, Iwamoto DK (2012). Heavy Drinking, Poor Mental Health, and Substance Use Among Asian Americans in the NLAAS: A Gender-Based Comparison. Asian Am J Psychol.

[66] Scott SB, Whitehead BR, Bergeman CS, Pitzer L (2013). Combinations of stressors in midlife: examining role and domain stressors using regression trees and random forests. J Gerontol B Psychol Sci Soc Sci.

[67] Cano A, Scaturo DJ, Sprafkin RP, Lantinga LJ, Fiese BH, Brand F (2003). Family Support, Self-Rated Health, and Psychological Distress. Prim Care Companion J Clin Psychiatry.

[68] Reardon SF, Fox L, Townsend J (2015). Neighborhood Income Composition by Household Race and Income, 1990–2009. Ann Am Acad Pol Soc Sci.

[69] South SJ, Huang Y, Spring A, Crowder K (2016). Neighborhood Attainment over the Adult Life Course. Am Sociol Rev.

[70] Sansone RA, Sansone LA (2010). Demoralization in patients with medical illness. Psychiatry (Edgmont).

[71] McNeill LH, Kreuter MW, Subramanian SV (2006). Social environment and physical activity: a review of concepts and evidence. Soc Sci Med.

[72] Hirotsu C, Tufik S, Andersen ML (2015). Interactions between sleep, stress, and metabolism: From physiological to pathological conditions. Sleep Sci.

[73] Meerlo P, Sgoifo A, Suchecki D (2008). Restricted and disrupted sleep: Effects on autonomic function, neuroendocrine stress systems and stress responsivity. Sleep Med Rev.

[74] Ulrich-Lai YM, Fulton S, Wilson M, Petrovich G, Rinaman L (2015). Stress exposure, food intake and emotional state. Stress Int J Biol Stress.

[75] Ridner SH (2004). Psychological distress: concept analysis. J Adv Nurs.

[76] Cruza-Guet MC, Spokane AR, Caskie GIL, Brown SC, Szapocznik J (2008). The Relationship Between Social Support and Psychological Distress Among Hispanic Elders in Miami. Florida J Couns Psychol.

[77] Rosenman R, Tennekoon V, Hill LG (2011). Measuring bias in self-reported data. Int J Behav Healthc Res.

[78] Do DP, Locklar LRB, Florsheim P (2019). Triple jeopardy: the joint impact of racial segregation and neighborhood poverty on the mental health of black Americans. Soc Psychiatry Psychiatr Epidemiol.

[79] Weden MM, Carpiano RM, Robert SA (2008). Subjective and objective neighborhood characteristics and adult health. Soc Sci Med.

[80] Johns LE, Aiello AE, Cheng C, Galea S, Koenen KC, Uddin M (2012). Neighborhood social cohesion and posttraumatic stress disorder in a community-based sample: findings from the Detroit Neighborhood Health Study. Soc Psychiatry Psychiatr Epidemiol.

[81] Bateman LB, Fouad MN, Hawk B, Osborne T, Bae S, Eady S (2017). Examining Neighborhood Social Cohesion in the Context of Community-based Participatory Research: Descriptive Findings from an Academic-Community Partnership. Ethn Dis.

[82] Sefcik JS, Kondo MC, Klusaritz H, Sarantschin E, Solomon S, Roepke A (2019). Perceptions of Nature and Access to Green Space in Four Urban Neighborhoods. Int J Environ Res Public Health.

