# Supplementary figures and images for: Neighborhood social cohesion and serious psychological distress among Asian, Black, Hispanic/Latinx, and White adults in the United States: a cross-sectional study
Source: BMC Public Health. 2022 Jun 15;22:1191. doi: 10.1186/s12889-022-13572-4 (PMC9199195; doi:10.1186/s12889-022-13572-4)

**Supplemental Figure 1. Composition of Analytic Sample**

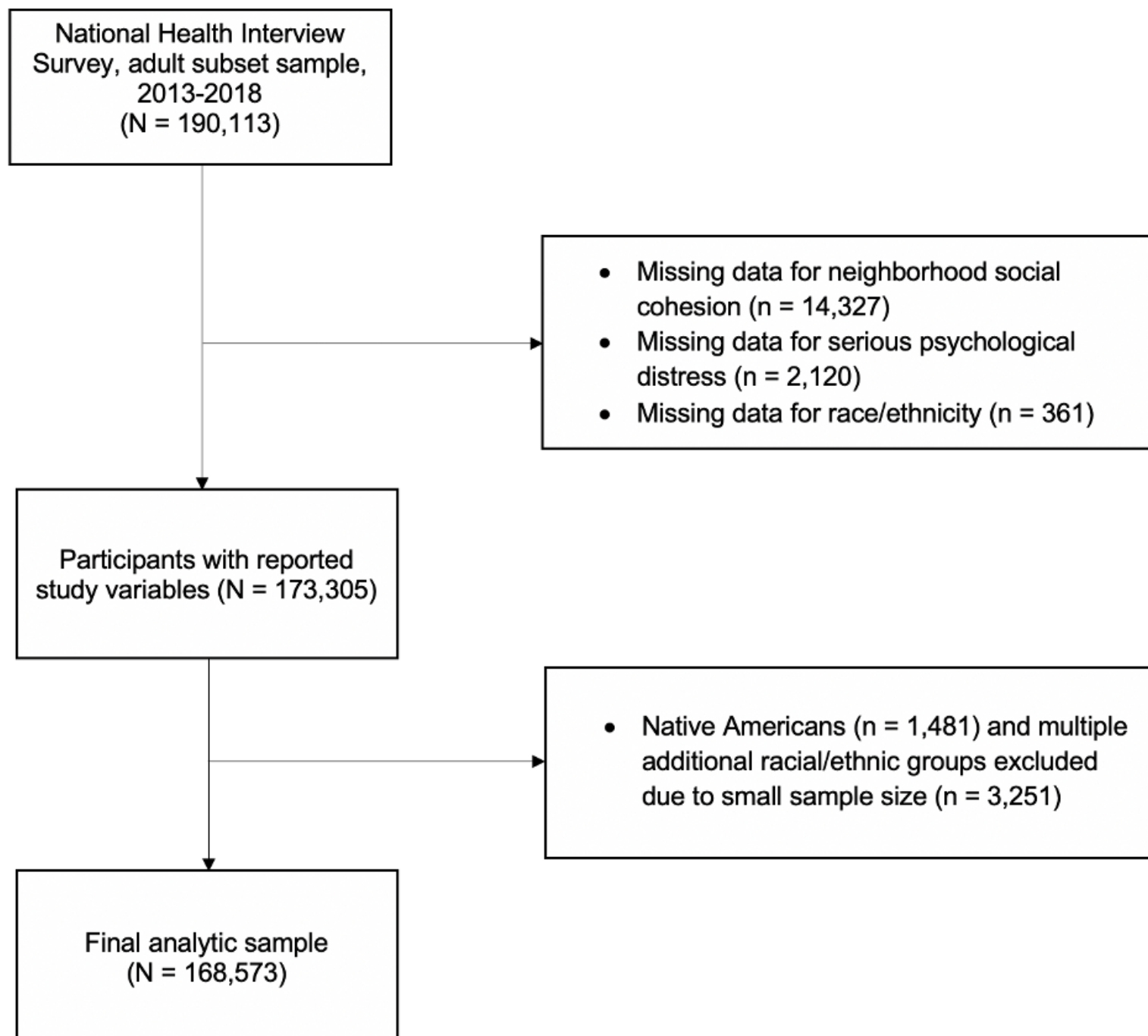

Supplement: Supplementary file 1 — Additional file 1: Supplemental figure 1. Composition of Analytic Sample. [file 12889_2022_13572_MOESM1_ESM.pdf]
