# Supplementary material for: Neighborhood social cohesion and serious psychological distress among Asian, Black, Hispanic/Latinx, and White adults in the United States: a cross-sectional study
Source: BMC Public Health. 2022 Jun 15;22:1191. doi: 10.1186/s12889-022-13572-4 (PMC9199195; doi:10.1186/s12889-022-13572-4)
Supplement: Supplementary file 2 — Additional file 2: Supplemental Table 1. Age-Standardized Sociodemographic Characteristics across components of the Neighborhood Social Cohesion Scale (N=168,573). [file 12889_2022_13572_MOESM2_ESM.pdf]

Supplemental Table 1. Age-standardized Sociodemographic Characteristics across components of the Neighborhood Social Cohesion Scale (N=168,573)

	Neighborhood Social Cohesion			
	**People in this neighborhood help each other out**	**There are people I can count on in this neighborhood**	**People in this neighborhood can be trusted**	**This is a close-knit neighborhood**
All	69158 (41.0%)	85426 (50.7%)	79128 (46.9%)	49875 (29.6%)
SPD	29.9%	37.6%	31.8%	22.6%
Race/ethnicity				
Asian	38.8%	43.1%	43.2%	28.5%
NH-Black	32.1%	38.4%	30.6%	26.4%
Hispanic/Latinx	30.9%	38.6%	34.9%	24.9%
NH-White	45.5%	57.1%	54.5%	31.6%
Sex/gender				
Women	42.3%	52.3%	48.7%	30.3%
Age				
18-30 years	30.7%	39.4%	35.8%	22.6%
31-49 years	38.9%	47.7%	43.9%	28.3%
≥50 years	45.5%	56.4%	53.7%	32.3%

Note. All estimates are weighted for the survey's complex sampling design. All estimates are age-standardized to the U.S. 2010 population, except for age.
SPD=Serious Psychological Distress
