# Supplementary material for: Neighborhood social cohesion and serious psychological distress among Asian, Black, Hispanic/Latinx, and White adults in the United States: a cross-sectional study
Source: BMC Public Health. 2022 Jun 15;22:1191. doi: 10.1186/s12889-022-13572-4 (PMC9199195; doi:10.1186/s12889-022-13572-4)
Supplement: Supplementary file 3 — Additional file 3: Supplemental Table 2. Prevelence Ratios of PD and SPD by nSC, Overall and Stratified by Race/Ethnicity, Sex/Gender (N=168,573). [file 12889_2022_13572_MOESM3_ESM.pdf]

Supplemental Table 2. Prevalence Ratios of PD and SPD by nSC, Overall and Stratified by Race/Ethnicity, Sex/Gender (N=168573)

	PR (95% CI)									
	**Overall N=168573**		**Asian N=9002**		**NH-Black N=20763**		**Hispanic/Latinx N=25235**		**NH-White N=113573**	
**Neighborhood Social Cohesion**	**PD[a*]**	**SPD[b*]**	**PD**	**SPD**	**PD**	**SPD**	**PD**	**SPD**	**PD**	**SPD**
	**All n=168573**									
Low vs. High	**1.54 (1.48-1.60)**	**1.91 (1.75-2.10)**	**1.72 (1.43-2.06)**	1.40 (0.71-2.78)	**1.47 (1.30-1.66)**	**1.55 (1.22-1.97)**	**1.42 (1.27-1.58)**	**1.85 (1.43-2.41)**	**1.55 (1.48-1.63)**	**1.96 (1.76-2.18)**
Medium vs. High	**1.31 (1.26-1.36)**	**1.18 (1.06-1.30)**	**1.39 (1.15-1.68)**	1.36 (0.73-2.54)	**1.25 (1.10-1.42)**	1.05 (0.81-1.35)	**1.25 (1.11-1.40)**	0.97 (0.74-1.28)	**1.31 (1.26-1.37)**	**1.26 (1.09-1.38)**
	**Men n=76051**									
Low vs. High	**1.49 (1.40-1.59)**	**2.01 (1.73-2.33)**	**1.70 (1.29-2.24)**	**4.88 (1.93-12.33)**	**1.56 (1.28-1.92)**	**1.65 (1.09-2.49)**	**1.33 (1.11-1.60)**	**1.71 (1.12-2.62)**	**1.50 (1.40-1.62)**	**2.03 (1.71-2.43)**
Medium vs. High	**1.32 (1.25-1.41)**	**1.21 (1.02-1.43)**	**1.72 (1.28-2.30)**	**3.81 (1.46-10.00)**	**1.36 (1.11-1.65)**	1.27 (0.84-1.93)	**1.24 (1.05-1.47)**	0.81 (0.50-1.32)	**1.31 (1.22-1.41)**	**1.26 (1.02-1.55)**
	**Women n=92522**									
Low vs. High	**1.57 (1.50-1.66)**	**1.87 (1.67-2.10)**	**1.74 (1.35-2.26)**	0.73 (0.31-1.73)	**1.41 (1.22-1.63)**	**1.50 (1.11-2.04)**	**1.47 (1.29-1.69)**	**2.00 (1.47-2.71)**	**1.59 (1.50-1.68)**	**1.93 (1.69-2.19)**
Medium vs. High	**1.29 (1.23-1.35)**	**1.16 (1.03-1.30)**	1.17 (0.90-1.52)	0.84 (0.39-1.80)	**1.18 (1.01-1.39)**	0.95 (0.68-1.34)	**1.24 (1.06-1.45)**	1.15 (0.86-1.55)	**1.31 (1.24-1.39)**	**1.21 (1.05-1.39)**

[a]PD (Psychological Distress) is Kessler 6-item Distress of 5-12
[b]SPD (Serious Psychological Distress) is Kessler 6-item Distress of 13-24
*Ref group is Kessler 6-item Distress of 0-4
PR=Prevalence Ratio; CI=Confidence Interval;
Adjusted for age (18-30, 31-49, ≥50 years), educational attainment (<high school, high school graduate, some college, ≥college), annual household income (<$35,000, $35,000-$74,999, $75,000+), occupational class (professional/management, support services, laborers), region of residence (Northeast, Midwest, South, West), alcohol consumption (never, former, current), "ideal" cardiovascular health (never smoking/quit >12 months prior to interview, BMI 18.5-<25 kg/m2, meeting physical activity guidelines, and no prior diagnosis of dyslipidemia, hypertension, or diabetes/prediabetes), marital/co-habiting status (married/living with partner or cohabitating, divorced/widowed/separated, single/no live-in partner), employment status (unemployed, employed), and self-rated health status (excellent/very good, good, fair/poor).
All model additionally adjusted for sex (woman, man).
Overall models adjusted for race/ethnicity
Note. All estimates are weighted for the survey's complex sampling design. Boldface indicates statistically significant results at the 0.05 level.
