# Supplementary material for: Neighborhood social cohesion and serious psychological distress among Asian, Black, Hispanic/Latinx, and White adults in the United States: a cross-sectional study
Source: BMC Public Health. 2022 Jun 15;22:1191. doi: 10.1186/s12889-022-13572-4 (PMC9199195; doi:10.1186/s12889-022-13572-4)
Supplement: Supplementary file 4 — Additional file 4: Supplemental Table 3. Prevelence Ratios of PD and SPD: Racial Minoritized Participants vs. NH-Whites with High nSC (N=168,573). [file 12889_2022_13572_MOESM4_ESM.pdf]

Supplemental Table 3. Prevalence Ratios of PD and SPD: Racial Minoritized Participants vs. NH-Whites with High nSC (N=168573)

	PR (95% CI)					
	Asian N=9002		NH-Black N=20763		Hispanic/Latinx N=25235	
**Neighborhood Social Cohesion**	**PD**	**SPD**	**PD[a*]**	**SPD[b*]**	**PD**	**SPD**
	**All n=168573**					
Low vs. NH-White High	**1.34 (1.19-1.51)**	1.02 (0.71-1.48)	**1.13 (1.05-1.23)**	0.96 (0.80-1.15)	**1.15 (1.07-1.25)**	**1.55 (1.30-1.84)**
Medium vs. NH-White High	1.12 (0.98-1.28)	0.96 (0.66-1.40)	1.00 (0.91-1.09)	**0.68 (0.54-0.84)**	1.02 (0.93-1.11)	**0.74 (0.59-0.93)**
High vs. NH-White High	**0.85 (0.72-0.99)**	0.74 (0.43-1.25)	**0.80 (0.71-0.90)**	**0.62 (0.49-0.79)**	**0.83 (0.74-0.93)**	0.85 (0.65-1.12)
	**Men n=76051**					
Low vs. NH-White High	**1.25 (1.05-1.49)**	1.72 (0.96-3.06)	**1.20 (1.06-1.36)**	1.08 (0.82-1.43)	1.12 (0.98-1.27)	**1.79 (1.37-2.33)**
Medium vs. NH-White High	**1.31 (1.08-1.58)**	1.41 (0.84-2.35)	1.06 (0.93-1.21)	0.88 (0.64-1.21)	1.06 (0.92-1.21)	0.76 (0.52-1.12)
High vs. NH-White High	0.80 (0.62-1.04)	**0.39 (0.17-0.87)**	**0.81 (0.67-0.98)**	**0.66 (0.45-0.98)**	0.87 (0.73-1.04)	1.03 (0.63-1.70)
	**Women n=92522**					
Low vs. NH-White High	**1.39 (1.19-1.62)**	0.75 (0.46-1.21)	**1.10 (1.00-1.21)**	0.92 (0.73-1.15)	**1.18 (1.07-1.30)**	**1.44 (1.14-1.80)**
Medium vs. NH-White High	0.96 (0.80-1.15)	0.74 (0.41-1.33)	0.95 (0.85-1.07)	**0.58 (0.43-0.78)**	1.00 (0.90-1.12)	**0.74 (0.56-0.97)**
High vs. NH-White High	0.88 (0.71-1.09)	0.90 (0.49-1.65)	**0.80 (0.69-0.92)**	**0.63 (0.48-0.84)**	**0.80 (0.70-0.93)**	0.74 (0.55-1.00)

[a]PD (Psychological Distress) is Kessler 6-item Distress of 5-12
[b]SPD (Serious Psychological Distress) is Kessler 6-item Distress of 13-24
*Ref group is Kessler 6-item Distress of 0-4
PR=Prevalence Ratio; CI=Confidence Interval;
Adjusted for age (18-30, 31-49, ≥50 years), educational attainment (<high school, high school graduate, some college, ≥college), annual household income (<$35,000, $35,000-$74,999, $75,000+), occupational class (professional/management, support services, laborers), region of residence (Northeast, Midwest, South, West), alcohol consumption (never, former, current), "ideal" cardiovascular health (never smoking/quit >12 months prior to interview, BMI 18.5-<25 kg/m2, meeting physical activity guidelines, and no prior diagnosis of dyslipidemia, hypertension, or diabetes/prediabetes), marital/co-habiting status (married/living with partner or cohabitating, divorced/widowed/separated, single/no live-in partner), employment status (unemployed, employed), and self-rated health status (excellent/very good, good, fair/poor).
All model additionally adjusted for sex (woman, man).
